# Unsupervised Domain Adaptation With Optimal Transport in Multi-Site Segmentation of Multiple Sclerosis Lesions From MRI Data

**DOI:** 10.3389/fncom.2020.00019

**Published:** 2020-03-09

**Authors:** Antoine Ackaouy, Nicolas Courty, Emmanuel Vallée, Olivier Commowick, Christian Barillot, Francesca Galassi

**Affiliations:** ^1^Empenn, INRIA, IRISA, CNRS, INSERM, Rennes, France; ^2^Panama/Obélix, INRIA, IRISA, Université de Bretagne Sud, Vannes, France; ^3^Orange Labs, Lannion, France

**Keywords:** MS lesion segmentation, deep learning, convolutional neural networks, unsupervised domain adaptation, optimal transport

## Abstract

Automatic segmentation of Multiple Sclerosis (MS) lesions from Magnetic Resonance Imaging (MRI) images is essential for clinical assessment and treatment planning of MS. Recent years have seen an increasing use of Convolutional Neural Networks (CNNs) for this task. Although these methods provide accurate segmentation, their applicability in clinical settings remains limited due to a reproducibility issue across different image domains. MS images can have highly variable characteristics across patients, MRI scanners and imaging protocols; retraining a supervised model with data from each new domain is not a feasible solution because it requires manual annotation from expert radiologists. In this work, we explore an unsupervised solution to the problem of domain shift. We present a framework, Seg-JDOT, which adapts a deep model so that samples from a source domain and samples from a target domain sharing similar representations will be similarly segmented. We evaluated the framework on a multi-site dataset, MICCAI 2016, and showed that the adaptation toward a target site can bring remarkable improvements in a model performance over standard training.

## 1. Introduction

Multiple Sclerosis (MS) is a chronic inflammatory-demyelinating disease of the central nervous system. Magnetic Resonance Imaging (MRI) is fundamental to characterize and quantify MS lesions; the number and volume of lesions are used for MS diagnosis, to track its progression and to evaluate treatments (Smith and McDonald, 1999). Current MRI protocols in MS consists in Fluid-Attenuated Inversion Recovery (FLAIR) and T1-weighted (T1-w) images, offering complementary contrasts that allows to identify different types of lesions. Accurate identification of MS lesions in MRI images is extremely difficult due to variability in lesion location, size, and shape, in addition to anatomical variability across patients. Since manual segmentation requires expert knowledge, it is time consuming and prone to intra- and inter-expert variability, several methods have been proposed to automatically segment MS lesions (García-Lorenzo et al., 2013; Commowick et al., 2018; Galassi et al., 2018).

In recent years, Convolutional Neural Networks (CNNs) have showed better performances in MS lesion segmentation than the traditional unsupervised methods (Commowick et al., 2018; Galassi et al., 2019). Yet, their clinical use remains limited due to a reproducibility issue across different sites or image domains. MRI MS imaging data can have high or subtle variations across individuals, MR scanners, and data acquisition protocols (Galassi et al., 2019; Kushibar et al., 2019; Onofrey et al., 2019). In research, the data used to train and test CNN models are never fully representative of all clinical scenarios, resulting in supervised models that suffer from poor generalization when applied to a new target image domain (Commowick et al., 2018).

A few studies have proposed methods to facilitate model re-training and re-use, such as Transfer Learning strategies (Kushibar et al., 2019), where the weights of an already trained network are tuned to adapt to a new target domain, decreasing the training time and demanding fewer training annotated samples than full training. Recent studies in computer vision propose Unsupervised Domain Adaptation strategies that do not require ground truth segmentation for the target dataset (Kouw and Loog, 2019). Our work deals with this more challenging and common scenario.

Unsupervised Domain Adaptation includes adversarial loss functions and adversarial image generation based methods (Sankaranarayanan et al., 2017; Tzeng et al., 2017). Generative adversarial approaches may generate image samples that are highly different from the actual MRI MS images and therefore make the network learn useless representations. One of the most recent works in Unsupervised Domain Adaptation proposes a solution for a classification task based on Optimal Transport, which learns a shared embedding for the source and target domains while preserving the discriminative information used by the classifier (Damodaran et al., 2018). Our framework is based on the latter approach. Learning a shared representation is suitable and relevant to our task where the aim is segmenting the same objects, MS lesions, within the same structure, the human brain.

In the sections that follow, we describe the use of Optimal Transport for Unsupervised Domain Adaptation and our original proposal, the Seg-JDOT framework. Seg-JDOT performs domain adaptation in a segmentation task thus alleviating the issue of low generalization ability in MS lesions segmentation. We demonstrate the effect of the adaptation on the classifier performance over standard training when training a model using data from a single site only and from multiple clinical sites. We employed the MICCAI 2016 dataset, which includes MRI MS images acquired with different scanners and protocols, and comprises patients with variable size and number of lesions.

## 2. Methods

### 2.1. Problem Statement

The problem of generalizing across domains can be formally defined. Let Ω ∈ ℝ be an input space of dimension *d*, C the set of labels, and P(Ω) the set of all probability measures over Ω. Let *X* be the instance space and *Y* the label space. The differences between domains can be characterized by a change in the marginal feature distributions P(X) and in the conditional distributions P(Y|X).

In standard learning for a classification task, one assumes the existence of a source dataset (**X**_*s*_, **Y**_*s*_), where ${\text{X}}_{s}\;=\;{\left\{{\text{x}}_{i}^{s}\right\}}_{i=1}^{N_{s}}$ is the instance data and ${\text{Y}}_{s}\;=\;{\left\{{\text{y}}_{\text{i}}^{s}\right\}}_{i=1}^{N_{s}}\in C$ is the corresponding class labels, and a target dataset ${\text{X}}_{t}\;=\;{\left\{{\text{x}}_{\text{j}}^{\text{t}}\right\}}_{j=1}^{N_{t}}$ with unknown labels **Y**_*t*_. To infer the labels on the target dataset, one learns an empirical estimate of the joint probability distribution P(X,Y)∈P(Ω×C) from (**X**_*s*_, **Y**_*s*_) by learning a classifier *f*, under the assumption that the source and target data are drawn from the same distribution μ∈P(Ω). However, if the target set is drawn from a slightly different distribution, the learned classifier might under-perform on the target set. If the drift between the two distributions is not too large, a domain adaptation approach can be used to improve learned model generalization.

In our work, we deal with a domain adaptation problem that assumes the existence of two distinct joint probability distributions, $P_{s}\left(X,Y\right)$ and $P_{t}\left(X,Y\right)$, corresponding respectively to the source domain and to the target domain, with respective marginal distributions μ_*s*_ and μ_*t*_ over Ω. We aim at leveraging the available information {**X**_*s*_, **Y**_*s*_, **X**_*t*_} to learn a classifier *f*, that is a labeling function $\hat{f}$ which approximates *f*_*s*_ and is closer to *f*_*t*_ than any other function ${\hat{f}}_{s}$. In order to solve this unsupervised domain adaptation problem, the Optimal Transport theory can be employed (Courty et al., 2017; Damodaran et al., 2018).

#### 2.1.1. Optimal Transport for Unsupervised Domain Adaptation

Optimal Transport is a theory that allows to compare and align probability distributions by seeking for a transport plan between them (Villani, 2008). Optimal Transport has been adopted in Unsupervised Domain Adaptation in order to compare the source and target distributions and bring them closer. Earlier use of Optimal Transport in Unsupervised Domain Adaptation involves finding a common latent space between the source and target domains where to learn a unique classifier, or finding a transport plan between the marginal feature distributions μ under the assumption of label regularity, i.e., the conditional probability remains unchanged (Gopalan et al., 2011; Courty et al., 2015).

Recently, Courty et al. proposed an approach that handles a shift in both the marginal and conditional probabilities, the Joint Distribution Optimal Transport framework (JDOT) (Courty et al., 2017). Formally, following the formulation of Optimal Transport given by Kantorovich (1942), their approach seeks for a transport plan between the two joint distributions $P_{s}$ and $P_{t}$, or equivalently a probabilistic coupling, γ ∈ Π$\left(P_{s},P_{t}\right)$ such that:

(1) $$\begin{array}{r} \gamma_{0}=\underset{\gamma \in \Pi \left(P_{s},P_{t}\right)}{\text{arg min}}\int_{\Omega \times \Omega }D(x^{s},y^{s};x^{t},y^{t})d\gamma (x^{s},y^{s};x^{t},y^{t}), \end{array}$$

where D is a joint cost function measuring both the dissimilarity between samples **x**^**s**^ and **x**^**t**^, and the discrepancy between **y**^**s**^ and **y**^**t**^. Because it is an unsupervised problem, the labels **y**^**t**^ are unknown and replaced by a proxy *f*(**x**^**t**^). Hence, they devised an efficient algorithm that aligns jointly the feature space and label-conditional distributions, by optimizing simultaneously for a coupling γ between $P_{s}$ and $P_{t}$ and a predictive function *f* embedded in the cost function. The classifier *f* on a target domain is learned according to the following optimization problem:

(2) $$\begin{array}{r} \underset{f,\gamma \in \Pi }{\min}\underset{ij}{\sum }D\left({\text{x}}_{i}^{s},{\text{y}}_{i}^{s};{\text{x}}_{j}^{t},f\left({\text{x}}_{j}^{t}\right)\right)\gamma_{ij}, \end{array}$$

where

(3) $$\begin{array}{r} D\left({\text{x}}_{i}^{s},{\text{y}}_{i}^{s};{\text{x}}_{j}^{t},f\left({\text{x}}_{j}^{t}\right)\right)=\alpha c\left({\text{x}}_{i}^{s},{\text{x}}_{j}^{t}\right)+\beta L\left({\text{y}}_{i}^{s},f\left({\text{x}}_{j}^{t}\right)\right) \end{array}$$

is a weighted combination of the distances in the feature space and the loss *L* in the label space, for the i-th source and the j-th target sample.

Two limitations can be identified in the JDOT framework: (i) the cost *c* is computed in the image space which can be poorly informative of the dissimilarity between samples, and (ii) the problem becomes intractable for large datasets since the coupling γ scales quadratically with the number of samples.

Subsequently, Damodaran et al. proposed a deep learning strategy to solve these two drawbacks (Damodaran et al., 2018). Their Deep-JDOT framework (i) minimizes the cost *c* in a deep layer of a Convolutional Neural Network, which is more informative than the original image space, and (ii) solves the problem with a stochastic approximation via mini-batches from the source and target domains. The Deep-JDOT model is thus composed of an embedding function *g* : **x** → **z** which maps the input space into a latent space, i.e., the output of a deep layer in the CNN, and a classifier *f* : **z** → **y** which maps the latent space into the output space. The optimization problem in Equation (2) therefore becomes:

(4) $$\begin{array}{r} \underset{\gamma \in \Pi ,f,g}{\min}\underset{ij}{\sum }D\left(g\left({\text{x}}_{i}^{s}\right),{\text{y}}_{i}^{s};g\left({\text{x}}_{j}^{t}\right),f\left(g\left({\text{x}}_{j}^{t}\right)\right)\right)\gamma_{ij}, \end{array}$$

where

(5) $$\begin{array}{l} D(g(x_{i}^{s}),y_{i}^{s};g(x_{j}^{t}),f(g(x_{j}^{t}))=\alpha ||g(x_{i}^{s})-g(x_{j}^{t}){||}^{2}\\ \;+\beta L_{t}(y_{i}^{s},f(g(x_{j}^{t}))). \end{array}$$

The first term in Equation (5) compares the embeddings for the source and the target domain, the second term considers the classification loss in the target domain and its regularity with respect to the labels in the source domain.

Equation (5) optimizes jointly the embedding function and the classifier to provide a model that performs well on a target domain. However, because Equation (5) takes into account the classifier learned in the target domain only, *f*(*g*(**x**^*t*^)), a performance degradation in the source domain might happen. To avoid such a degradation, they reintroduce the loss function *L*_*s*_ evaluating the classifier learned on the source domain, *f*(*g*(**x**^*s*^)), yielding the following optimization problem:

(6) $$\begin{array}{l} \underset{\gamma ,f,g}{\min}\frac{1}{n^{s}}\sum_{i}L_{s}(y_{i}^{s},f(g(x_{i}^{s})))+\sum_{i,j}\gamma_{ij}(\alpha ||g(x_{i}^{s})-g(x_{j}^{t}){||}^{2}\\ \;+\beta L_{t}(y_{i}^{s},f(g(x_{j}^{t})). \end{array}$$

With this formulation, the framework learns a common latent space that conveys information for both the source and target domain. The final objective of Deep-JDOT is then to find an embedding function *g* (which is equivalent to finding a latent space **z**), a classifier *f* and a transportation matrix such that inputs from the source and target domains that are similar in the latent space **z** are similarly classified. Importantly, solving the optimization problem with a stochastic approximation yields a computationally feasible solution which can be easily integrated into a deep learning framework. This approach is the starting point of our work and it will be further recalled and detailed in the next sections.

### 2.2. The Seg-JDOT Framework

**Figure 1:**
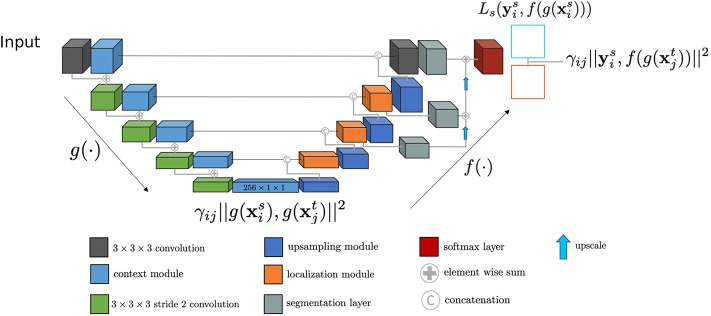
The Seg-JDOT framework. *g*(·) is the embedding function, *f*(·) is the segmenter and, in between them, the bottleneck representation is the latent space ***z*** where we perform the adaptation. We report the terms of the Equation (8) at the levels where they are applied. We represent the output source images with a blue square and the output target images with a red square.

We designed the Seg-JDOT framework to perform simultaneously a segmentation and an adaptation task. An overview of the framework is illustrated in Figure 1.

We employed a state-of-the-art deep learning architecture for brain lesion segmentation, a 3D-Unet (Isensee et al., 2018). The architecture was presented at the MICCAI BRATS 2018 segmentation challenge as an optimization of the original 3D-Unet proposed by Ronneberger et al. (2015).

The downward *context pathway* is a succession of *context modules*, with each module comprising two convolutional layers. The upward *localization pathway* combines the deepest representation with spatial information, brought by skip connections. This is achieved by first up-sampling the low dimensional representation and then combining it with the features from the corresponding output of the *context pathway*. To obtain the final segmentation maps, three different feature maps are combined through element-wise summation. Hence, from a compact representation with a low spatial dimension, a segmentation map with the same dimension as the input is obtained.

The model is composed of an embedding function *g* : *x* → *z*, which maps the input **x** into the bottleneck representation **z**, and a segmenter *f* : *z* → *y*, which maps the latent space **z** into the segmentation space **y**. Seg-JDOT optimizes jointly the latent space and the segmenter to provide a model that performs well on a target domain. In the sections that follow we provide a thorough description of the framework and the solution to the optimization problem.

#### 2.2.1. Defining the Probability Distributions and the Representation Space

As described in the previous section, Optimal Transport allows to align the probability distribution in the source domain, μ_*s*_, and the probability distribution in the target domain, μ_*t*_. Defining the two probability distributions and the space where to compute their coupling γ is not trivial and needs attention.

In a statistical context, we hardly have access to the true distribution μ; instead, we work with an empirical distribution ${\hat{\mu }}_{n}$. The number of samples *n* needed for ${\hat{\mu }}_{n}$ to be a reasonable proxy of μ grows with the number of dimensions *d* of the space in which the distribution lies, a limit known as *the curse of dimensionality* (Bellman, 1961). The Wasserstein distance can be used to quantify the convergence of ${\hat{\mu }}_{n}$ to μ. Dudley (1969) showed that μ absolutely continuous with respect to the Lebesgue measure on ℝ^*d*^ satisfies

(7) $$\begin{array}{r} 𝔼\left[W_{1}\left(\mu ,{\hat{\mu }}_{n}\right)\right]≲n^{-1/d} \end{array}$$

when *d* > 2. Equation (7) indicates that the expectation of the Wasserstein distance between ${\hat{\mu }}_{n}$ and μ grows exponentially with the number of dimensions *d*, a critical aspect in defining the probability distributions to be aligned.

In our work, we compute the Optimal Transport coupling in a deep layer of the CNN where the representation is compact and rich, which is the bottleneck layer of the 3D-Unet. The use of a compact latent space **z** allows to greatly reduce the original input dimensions. Moreover, solving the problem using mini-batches acts as a regularizer, which is important when working in high dimension (Genevay et al., 2019).

In order to define the probability distributions, we employ image patch samples rather than image samples as in Damodaran et al. (2018). The use of image patches enables an higher number of samples and, therefore, a more precise estimation of the true distribution μ. Indeed, five image samples per domain would be insufficient to adequately represent a distribution in **z**. It is important to notice that aligning patches rather than images is more reasonable for our task: two patches having similar lesions do not necessarily share the same location within the brain anatomy.

#### 2.2.2. Defining the Global Loss Function

Damodaran et al. designed the Deep-JDOT framework to solve a classification and adaptation task simultaneously (Damodaran et al., 2018), so that samples from the source and target domain having similar representations in the latent space will be similarly classified by the network. The assumption is that if two images share the same label then they should have similar, if not equal, activation maps at some depth in the network. In their work, the loss functions *L*_*s*_ and *L*_*t*_ in Equation (6), respectively the loss in the label space in the source and in the target domain, were chosen to be the same i.e., the cross-entropy.

In our segmentation task, however, the correspondence between two similar activation maps and two similar segmentation maps is harder to establish. The variety of segmentation maps is generally much higher than the number of classes in a classification task. We cannot expect exact correspondence both in the latent space and in the segmentation space. While we chose the Dice Score as loss *L*_*s*_, the choice of the loss *L*_*t*_ was not trivial.

In order to define *L*_*t*_, we conducted experiments involving the use of the Dice Score and the Squared Euclidean Distance. Results indicated an improved network performance in completing the task when using the Squared Euclidean Distance. Results involving the use of the Dice score can be found in Supplementary Material. This behavior might be explained by the fact that if two patches comprise a lesion of similar size and shape but different location within the patch, the Dice Score computed in the output space might be low because sensitive to a lesion location. On the contrary, the distance $||g\left({\text{x}}_{i}^{s}\right)-g\left({\text{x}}_{j}^{t}\right){||}^{2}$ computed at the bottleneck layer of the network, where there is no spatial information, might indicate that the two representations are similar. Yet, for the framework to perform correctly the segmentation and adaptation task simultaneously, there must be an agreement between the distance in the latent space, *c*, and the loss in the output space, *L*_*t*_. The Squared Euclidean distance is less sensitive to a lesion location than the Dice Score and therefore more appropriate for our task. On the basis of such considerations, we formulated the global loss function as:

(8) $$\begin{array}{l} \underset{\gamma ,f,g}{\min}\frac{1}{n^{s}}\sum_{i}L_{s}(y_{i}^{s},f(g(x_{i}^{s})))+\sum_{i,j}\gamma_{ij}(\alpha ||g(x_{i}^{s})-g(x_{j}^{t}){||}^{2}\\ \;+\beta ||y_{i}^{s}-f(g(x_{j}^{t})){||}^{2}). \end{array}$$

#### 2.2.3. Learning With Seg-JDOT

In Equation (8) two groups of variables need to be optimized: the optimal transport matrix γ and the functions *g* and *f* induced by the network. As suggested by Courty et al., the problem can be addressed by alternatively solving Equation (8) for γ, with fixed *g* and *f*, and computing *g* and *f*, with fixed γ (Courty et al., 2017). When fixing ĝ and $\hat{f}$, solving Equation (8) is equivalent to solving a classic Optimal Transport problem with cost matrix $C_{ij}=\alpha ||ĝ\left({\text{x}}_{i}^{s}\right)-ĝ\left({\text{x}}_{j}^{t}\right){||}^{2}+\beta ||{\text{y}}_{i}^{s}-\hat{f}\left(ĝ\left({\text{x}}_{j}^{t}\right)\right){||}^{2}$; similarly, when fixing $\hat{\gamma }$, solving for *g* and *f* is a standard deep learning problem.

Damodoran et al. proposed to solve this optimization problem with a stochastic approximation using mini-batches from the source and target domains, so to ease the computation of the Optimal Transport (Damodaran et al., 2018). Using a mini-batch of size *m* leads to the following optimization problem:

(9) $$\begin{array}{l} \underset{f,g}{\text{min}}𝔼[\frac{1}{m}\sum_{i=1}^{m}L_{s}(y_{i}^{s},f(g(x_{i}^{s})))+\underset{\gamma \in \Gamma (\mu_{s},\mu_{t})}{\text{min}}\;\sum_{i,j=1}^{m}\gamma_{ij}(\alpha ||g(x_{i}^{s})\\ \;-g(x_{j}^{t}){||}^{2}+\beta ||y_{i}^{s}-f(g(x_{j}^{t})){||}^{2})], \end{array}$$

with 𝔼 the expected value with respect to the mini-batches from the source and target domains. We summarize this approach in Algorithm 1.

**Table:** **Algorithm 1**. Seg-JDOT stochastic optimization

**Require: x**^*s*^: source domain images, **x**^*t*^: target domain images, **y**^*s*^: source domain segmentation maps
**for** each source batch $\left({\text{x}}_{b}^{s},{\text{y}}_{b}^{s}\right)$ and target batch (${\text{x}}_{b}^{s}$) **do**
fix $\hat{g}$ and $\hat{f}$, find γ for the given batch
fix $\hat{\gamma }$, and use gradient descent to update $\hat{f}$ and $\hat{g}$
**end for**

In order to implement Algorithm 1, we separated the global loss function in Equation (9) into two loss functions that are computed at two different levels of the network.

We name the first loss function *representation alignment loss function* and compute it at the output of the bottleneck layer:

(10) $$\begin{array}{r} \overset{m}{\underset{i,j=1}{\sum }}\gamma_{ij}\alpha ||g\left({\text{x}}_{i}^{s}\right)-g\left({\text{x}}_{j}^{t}\right){||}^{2}. \end{array}$$

The *representation alignment loss function* ensures that a source sample and a target sample that are heavily connected (high γ value) have representations not far in the Euclidean distance sense. By back-propagating through all the shallower layers, we ensure a domain independent representation.

We name the second loss function *segmentation alignment loss function* and compute it at the final output layer:

(11) $$\begin{array}{r} \frac{1}{m}\overset{m}{\underset{i=1}{\sum }}L_{s}\left({\text{y}}_{i}^{s},f\left(g\left({\text{x}}_{i}^{s}\right)\right)\right)+\overset{m}{\underset{i,j=1}{\sum }}\gamma_{ij}\beta ||{\text{y}}_{i}^{s}-f\left(g\left({\text{x}}_{j}^{t}\right)\right){||}^{2}. \end{array}$$

The first term of the *segmentation alignment loss function* allows to avoid a degradation of the performances in the source domain; the second term ensures that a source sample connected to a target sample has an output which is not too far from the true segmentation of the target sample in the Euclidean distance sense.

## 3. Experiments and Results

### 3.1. Dataset

Proper selection of the dataset for the unsupervised domain adaptation experiments is crucial because the domain difference should be present to confirm the framework's robustness. In this work, we employ a well-known dataset, the MICCAI 2016 MS lesion segmentation challenge dataset (Commowick et al., 2018). It contains 53 MRI images of patients suffering from MS, split into 15 train and 38 test images. For each patient, high quality segmentation maps are provided—they were computed from seven independent manual segmentations and using LOPSTAPLE (Akhondi-Asl et al., 2014) so to minimize inter-expert variability.

Images were acquired in four different clinical sites, corresponding to four different MRI scanner models (Table 1). Each clinical site includes 5 train and 10 test patients (sites 01, 07, 08), except one site that contains 8 test patients only (site 03). In our experiments, we used the test images for testing purpose only and we never included them in the training or validation or adaptation process.

**Table 1:** The MICCAI 2016 MS lesion segmentation challenge dataset contains MR images of MS patients from four different MRI scanners.

**Site**	**MRI scanner**	**Modality**	**Train subjects**	**Test subjects**
01	GE Discovery 3T	3D FLAIR 3D T1	5	10
03	Philips Ingenia 3T	3D FLAIR 3D T1	0	8
07	Siemens Aera 1.5T	3D FLAIR 3D T1	5	10
08	Siemens Verio 3T	3D FLAIR 3D T1	5	10
Total			15	38

*Sites 01, 07, and 08 include 5 train images and 10 test images; site 03 contains 8 test images*.

**Figure 2:**
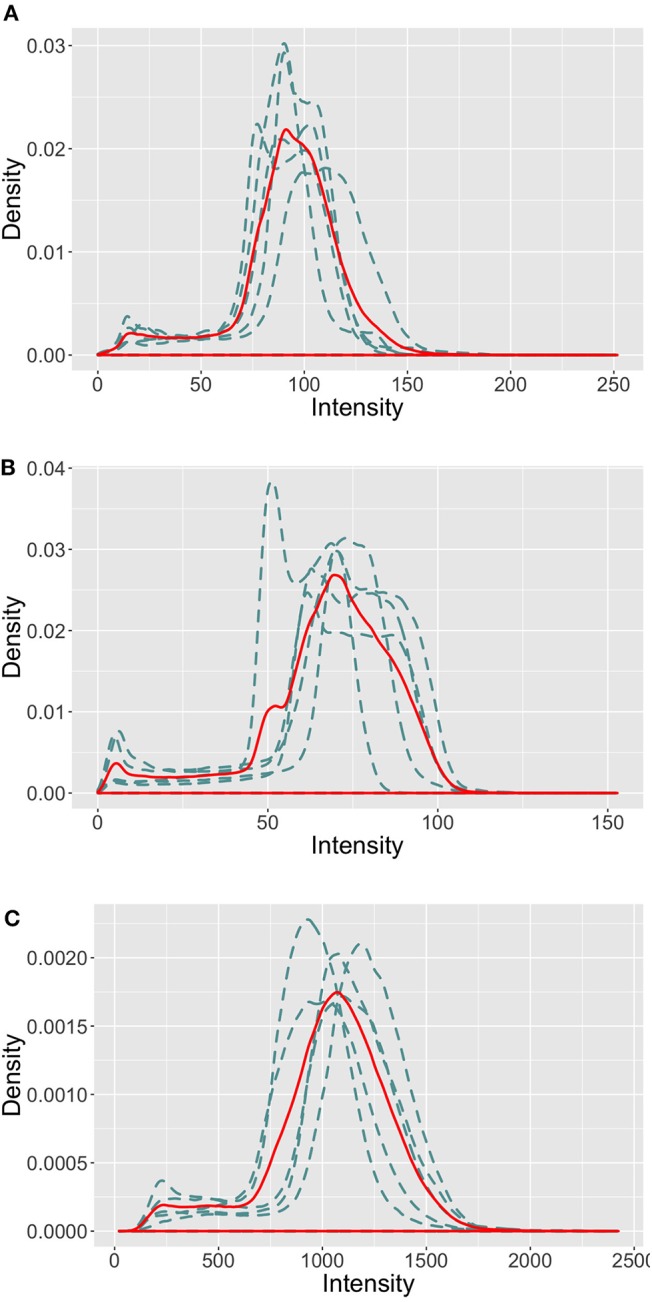
Intensity profiles in the brain area of the FLAIR images in the MICCAI 2016 train set. The blue dashed line represents the intensity distribution of each image, the red solid line represents the mean intensity distribution of the site images. **(A)** Site 01. **(B)** Site 07. **(C)** Site 08.

All MRI imaging protocols included 3D FLAIR and 3D T1-w anatomical images. Image size and resolution were different across the four MRI scanners (more details on the imaging protocol are available on the challenge website^1^). As illustrated in Figure 2, the intensity profiles in the brain area vary across the MRI scanners. Sites 01 and 07 follow a similar profile with a maximum intensity ≈ 200, while they vary drastically from site 08, where the intensity reaches up to ≈ 2,000 (a similar distribution was observed for site 03, test images). This behavior in intensity distribution was observed for both the imaging modalities, train and test patients.

**Figure 3:**
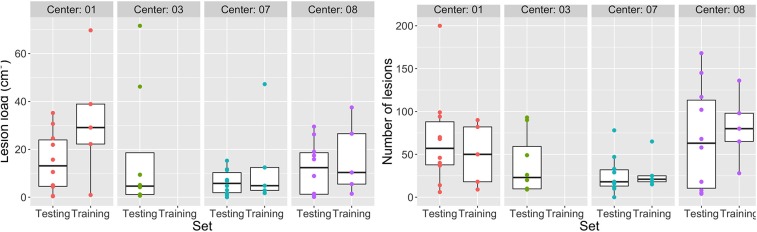
Variability in MS lesion volume and number. Lesion load per patient per site **(Left)** and Number of lesions per patient per site **(Right)**.

Moreover, patients show a variability in MS lesion volume and number of lesions (Figure 3). The median lesion load in the train (test) dataset is for site 01 ≈ 30(≈ 16)cm^3^, for site 03 ≈ (5)cm^3^, for site 07 ≈ 5(6)cm^3^, and for site 08 ≈ 10(12)cm^3^. A similar variation across sites was observed in the number of lesions.

Considering these variations across the four clinical sites, the MICCAI 2016 dataset does fit the challenge of the domain shift problem.

### 3.2. Implementation Details

#### 3.2.1. Image Pre-processing

Before extracting the patch samples from the image volumes to train the network, we performed a few standard pre-processing steps on the raw MRI images. For each patient, (i) MRI images were denoised (Coupe et al., 2008), (ii) rigidly registered toward the FLAIR modality (Commowick et al., 2012), (iii) skull-stripped (Manjón and Coupé, 2016), and (iv) bias corrected (Tustison et al., 2010). These steps involved the use of Anima, an openly available toolkit for medical image processing developed by the Empenn research team, Inria Rennes^2^.

In order to preserve the challenge of the domain shift, we did not standardize intensities across sites. However, as the drastic variation in the intensity profiles would make the training process unnecessarily hard, we adjusted the intensities of each patient image to have zero mean and unit variance.

#### 3.2.2. CNN Training

Images were resampled to the same size 128 × 128 × 128; 3D patches of size 16 × 16 × 16 were extracted. We employed a patch overlap of 50%, resulting in 4,096 patches per image. Although overlapping 3D patches contain more surrounding information for a voxel, it is memory demanding; training on patches containing lesions allowed to reduce training time while reducing class imbalance.

CNN training was performed in batches containing 256 source and 256 target samples, with a total batch size of 512—the maximum size that the employed GPU can handle. Since the quality of approximation of the true optimal transport coupling depends on the number of samples, we chose to use the maximum batch size possible.

#### 3.2.3. Technical Details

The Seg-JDOT framework was implemented in Python using the Keras library and the POT library (Flamary and Courty, 2017) which contains helpful functions for the Optimal Transport solver. Experiments were conducted on the GPU NVIDIA Quadro P6000, 24 GB.

### 3.3. Results on the MICCAI 2016 Dataset

We evaluated the segmentation performance when training both on a single site and on multiple clinical sites. The first experiment represents the worst case scenario, with training data acquired on a unique MR scanner; the second experiment reflects a more recurrent situation in the real practice, with training data coming from more than one MR scanner and a model that shall be more robust to variability.

#### 3.3.1. Single-Site Training

First, we evaluated the segmentation performance when training on a single site only. Hence, we applied the Seg-JDOT framework with one site as the source domain and any other site as the target domain. We did not perform adaptation toward the site 03 because it does not contain a train dataset.

The segmentation performance was assessed in terms of Dice score and F1 score. The Dice score is a measure of spatial overlap between the output and the ground truth; the F1 score is a weighted average of the lesion sensitivity and the positive predictive value, hence a metric that is independent of the lesion contour quality.

For each combination source/target, we compared the scores as obtained with the standard training (source only) with the scores as obtained with the adapted model. While the main focus of our study is the variation in performance on the target domain, we also evaluated the scores achieved by the adapted classifier on the other clinical sites. This allowed us to assess a possible degradation in the source domain performance and the overall effect of the adaptation on the model generalization ability.

**Figure 4:**
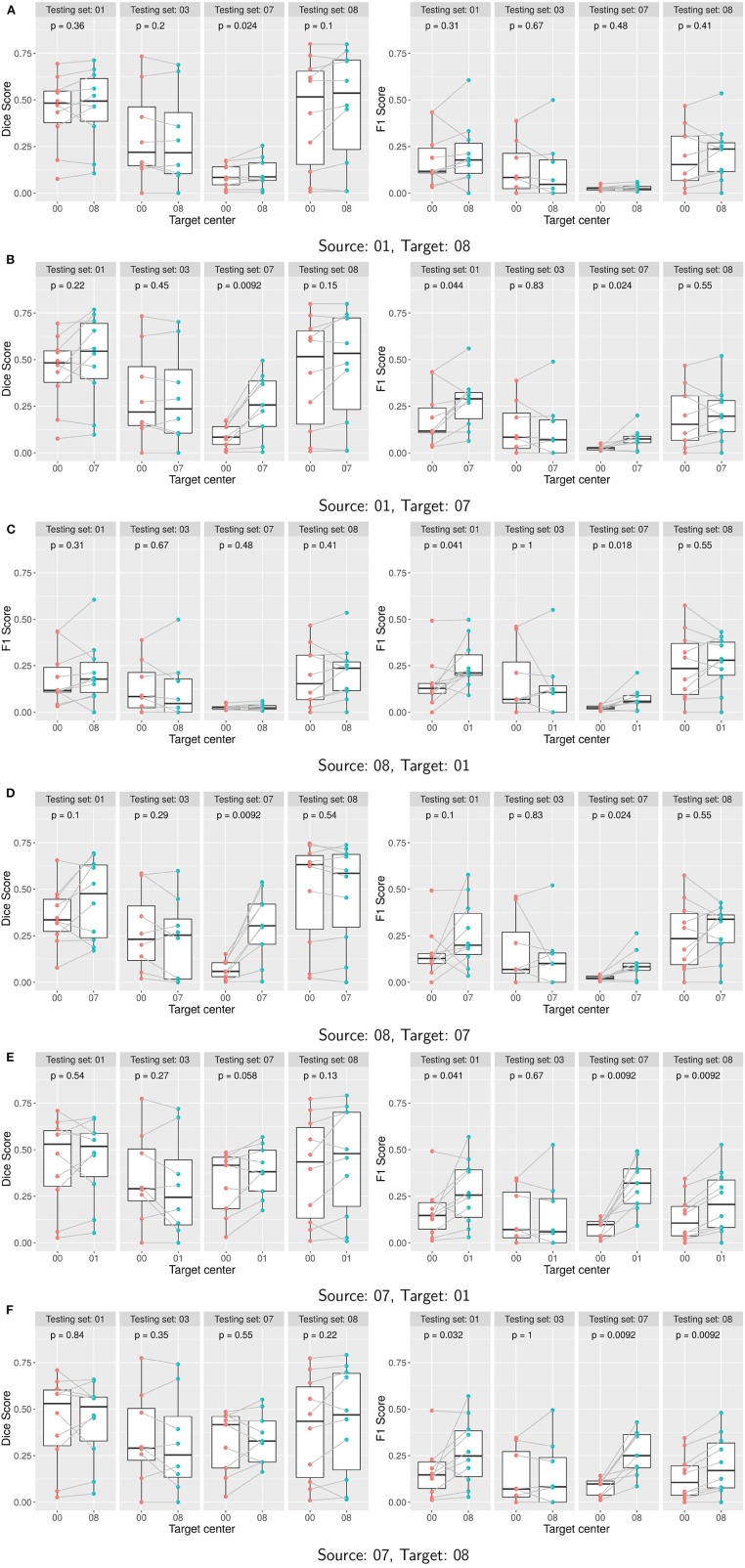
**(A–F)** Performance of Seg-JDOT with single-site source and single-site target domain adaptation. Each row corresponds to a combination of source and target. Dice score (left column) and F1 score (right column) are computed with no adaptation (00) and with Seg-JDOT, where the direction of the domain adaptation is indicated (07, 08, or 01). For each combination of source and target, performances are given for all the four testing sites. Each point is a patient of a given site; performances of a patient *with* and *without* Seg-JDOT are tracked. For each site, the *p*-value of the paired Wilcoxon test is reported.

Boxplots of the Dice and F1 scores (Figure 4) illustrate the effect of the domain adaptation. For each site, we assessed the significance between pair-wise comparisons of the performances of the two learned classifiers. The Shapiro-Wilk's test of normality indicated a non-normal distribution of the samples and thus a paired Wilcoxon test was used (Rey and Neuhäuser, 2011). Reported *p*-values were computed using the paired Wilcoxon test and indicate whether the variations are statistically significant: if the *p*-value is lower than the significance level of 0.05, then we can state that the scores as computed with the two approaches are significantly different.

**Figure 5:**
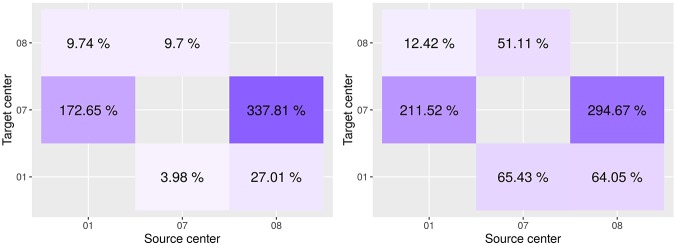
Variation in performance on the target site between the model as learned on the source only and adapted on the target domain. Dice score on the left, F1 score on the right. On the *x*-axis is the source center, on the *y*-axis is the target center.

In Figure 5, we report the overall percentage of variation in performance on the target site. A positive variation indicates an improvement in the score. More detailed information can be found in Supplementary Material.

Results indicate that target site performances generally improve when applying the Seg-JDOT framework. The domain adaptation toward the site 07 yields the most significant improvement in target performance (Figures 4B,D), while the adaptation toward the site 08 yields minor variations only (Figures 4A,F).

The highest improvement is registered for the combination source site 08 and target site 07 (Figure 5), with a variation in the Dice score and F1 score of about 338 and 295%, respectively. It indicates that the adaptation reduces the effect of the high variability in intensity and lesion load/number that we observed across the two sites. When considering the adaptation in the other direction, i.e., the combination source site 07 and target site 08, we observe that the variability across the two sites did not affect that much the model performance, with a variation in the Dice score and F1 score of about 10 and 51%, respectively. In other words, the model learned on the site 07 appears to be more robust and to generalize better to other sites. This might be due to the fact that the samples within the site 07 are the most challenging and representative among all the sites.

Adapting toward a target domain appears beneficial, or otherwise not detrimental, for the overall generalization ability of a model. For instance, for the combination source site 08 and target site 01 we note a significant improvement in segmentation outcome also on the test site 07 (Figure 4C). For the combination source site 01 and target site 08, the adaptation does not yield a significant improvement in performance on the target site (Figure 4A); yet, a minor improvement in the Dice score is registered on the test site 07. This suggests that the adaptation toward a target domain allows to learn a classifier that is less specific to the source domain and thus capable to generalize better.

The adaptation can be beneficial for the source site as well. We observe an improvement in the F1 score on the source site for the combination source site 07 and target site 01 (Figure 4E) or target site 08 (Figure 4F), and for the combination source site 01 and target site 07 (Figure 4B). This might be explained by the fact that the network is trained to minimize the Dice Score rather than the F1 Score and, therefore, the adaptation may move the network away from the optimal Dice Score solution and closer to the optimal F1 Score solution.

**Figure 6:**
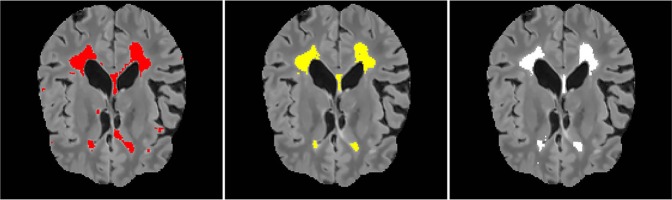
A qualitative result for the combination source site 08 and target site 07. The results are shown in the coronal views of the FLAIR image. From the left: a segmentation result on site 07 when training on the site 08, segmentation result after adaptation, ground truth.

A qualitative result on a patient from the site 07 for the combination source site 08 and target site 07 is shown in Figure 6. We observe that the adaptation toward the site 07 yields a better segmentation output than training on the source site only. The number of false positives appears greatly reduced.

#### 3.3.2. Multi-Site Training

We evaluated the segmentation performance when training on multiple clinical sites. Hence, the source domain comprised multiple sites (two) and the target domain was the remaining one. The site 03 was used for testing purpose only since it does not include a train dataset.

As for single-site training, the classifier performance was assessed in terms of Dice score and F1 score. For each combination source/target, we tested the classifier as adapted with Seg-JDOT on the target site as well as on the other test sites, so to assess the impact of the adaptation on the source performance and on the overall model generalization ability.

**Figure 7:**
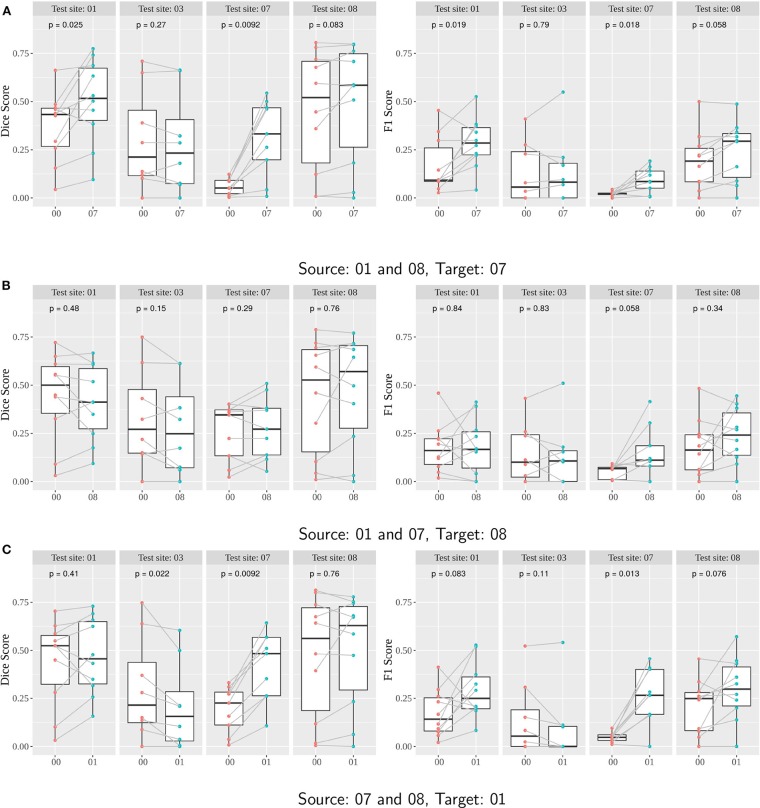
**(A–C)** Performance of Seg-JDOT with multi-site source and single-site target domain adaptation. Each row corresponds to a combination of source and target. Dice score (left column) and F1 score (right column) are computed with no adaptation (00) and with Seg-JDOT, where the direction of the domain adaptation is indicated (07, 08, or 01). For each combination of source and target, performances are given for all the four testing sites. Each point is a patient of a given site; performances of a patient *with* and *without* Seg-JDOT are tracked. For each site, the *p*-value of the paired Wilcoxon test is reported.

Boxplots of the Dice and F1 scores illustrate the effect of the domain adaptation on a clinical site (Figure 7). *P*-values were computed using the paired Wilcoxon-test.

**Figure 8:**
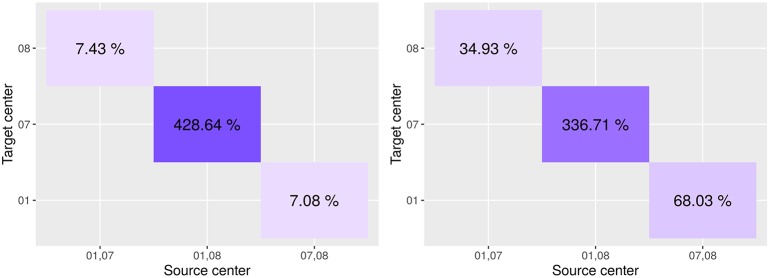
Variation in performance on the target site between the model as learned on the source only and adapted on the target domain. On the *x*-axis is the source center, on the *y*-axis is the target center.

In Figure 8, we report the overall percentage of variation in performance on a target site. A positive variation indicates an improvement in the score. More detailed information can be found in Supplementary Material.

Results indicate that Seg-JDOT generally improves the performances on the target site. As for single-site training, the most significant improvement is achieved on the target site 07 when the site 08 is a source domain (Figure 7A), with an overall variation in the Dice score of about 429% and in the F1 score of about 337% (Figure 8), while the least significant improvement is achieved on the target sites 08 (Figure 7B) and 01 (Figure 7C). This suggests that the less a model generalizes to a site, the more likely the adaptation will improve its performance on the latter, and vice-versa.

The adaptation can be beneficial for a source domain as well. We observe an improvement in the scores on the source site 01 for the combination source sites 01 and 08, and target site 07 (Figure 7A). Similarly, the source site 07 benefits from an adaptation toward the target site 01 (Figure 7C). For these combinations, the adaptation has thus a regularizing effect that yields an improvement in performance also on the source site.

In order to fully appreciate the effectiveness of the adaptation, we compared Seg-JDOT with training on standardized images. The intensities were standardized using the method of Nyul et al. (2000). Detailed results can be found in the Supplementary Material. A significant improvement was still achieved on the target site 07, with an overall variation in the Dice score of about 181% and in the F1 score of about 204%.

## 4. Discussion and Conclusion

In this paper, we presented the Seg-JDOT framework for Unsupervised Domain Adaptation based on Optimal Transport. The framework aims at adapting a model so that samples from a source and a target domain sharing similar representations will yield similar predictions. The framework was designed to perform an MS lesion segmentation task while addressing the recurrent situation of deploying a model on a clinical target site that was not included in the training process. Importantly, the adaptation does not require any manually annotated image in the target domain.

We tested the framework on the MICCAI 2016 MS lesion segmentation challenge dataset which includes four clinical sites presenting variations in intensity profile and lesion load or number. Our results with single-source and multi-source training indicate that the adaptation toward a target site can yield significant improvement in the model performance over standard training. The improvement appears to be the most significant for models having otherwise a low generalization ability. Adaptation toward a target site can bring improvements in the overall generalization ability of the model toward any domains. Also, the source performance is either not affected by the adaptation or an increase in the scores is observed.

A comparison of Seg-JDOT performances with training on standardized images indicates that the domain shift problem is still there after image standardization. This suggests that Seg-JDOT implicitly performs a normalization by adapting the weights to better interpret the features extracted by the network.

Although the approach was shown to be effective to deal with the domain adaptation problem, our dataset included clinical sites comprising five training subjects only. Future work will consider the evaluation of this approach with different data splits, other MS dataset and more subjects. Also, other measures of variability across sites and patients might be taken into account, such MS lesion types or patient age.

Seg-JDOT can easily be adapted to other neural network architectures or tasks. In this work, we have employed a variation of a 3D-Unet architecture recently proposed for a brain lesion segmentation task. However, the use of image-wise segmentation outputs, rather than voxel-wise, may limit the performance of the framework because the output predictions in the target domain can only approximately fit the target lesion. Future work will consider the evaluation of the framework with other CNN architectures, such as the voxel-wise CNN network proposed by Valverde et al. (2017).

## Data Availability Statement

Publicly available datasets were analyzed in this study. This data can be found here: https://portal.fli-iam.irisa.fr/msseg-challenge/data.

## Author's Note

This article has been released as a Preprint at Ackaouy et al. (2019).

## Author Contributions

AA conception and design of the work, code implementation and experiments, results interpretation, drafting the article, critical revision of the article. NC theoretical formalism, results interpretation, critical revision of the article. EV results interpretation, drafting the article, critical revision of the article. OC and CB conception and design of the work, data selection. FG conception, design and supervision of the work, data selection, results interpretation, drafting the article, critical revision of the article, final approval of the version to be published.

### Conflict of Interest

EV was employed by Orange Labs for a period of time, but is no longer affiliated. The company had no influence on or contribution to the design, methodology or results of this study. The remaining authors declare that the research was conducted in the absence of any commercial or financial relationships that could be construed as a potential conflict of interest.

## Footnotes

^1^https://portal.fli-iam.irisa.fr/msseg-challenge/data

^2^https://github.com/Inria-Visages/Anima-Public
