# Supplementary material for: Unsupervised Domain Adaptation With Optimal Transport in Multi-Site Segmentation of Multiple Sclerosis Lesions From MRI Data
Source: Front Comput Neurosci. 2020 Mar 9;14:19. doi: 10.3389/fncom.2020.00019 (PMC7075308; doi:10.3389/fncom.2020.00019)
Supplement: Supplementary file 1 [file Data_Sheet_1.PDF]

Supplementary Material

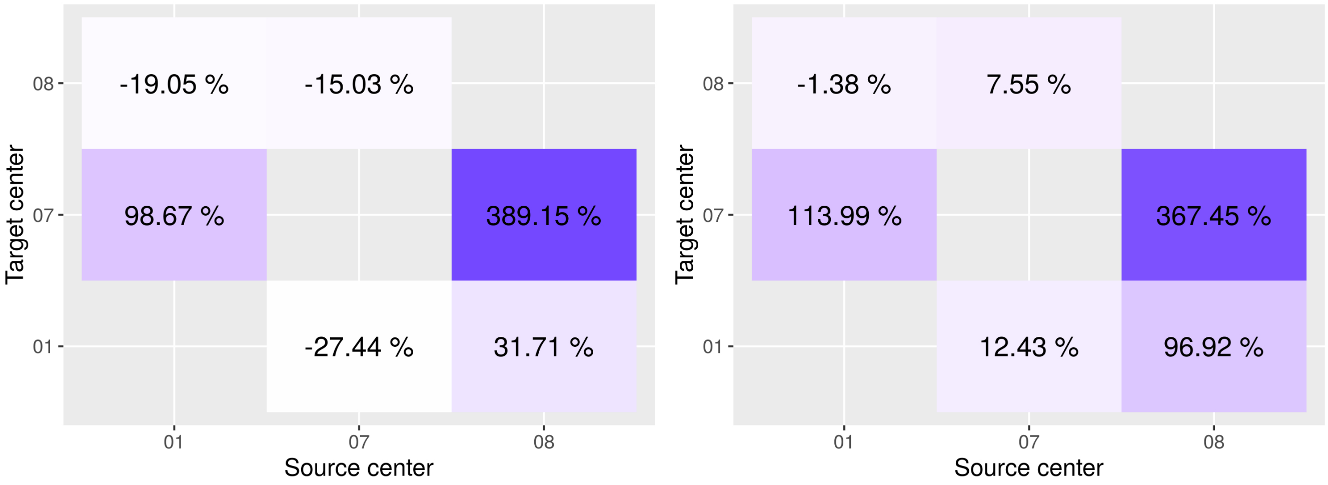

Figure S1. Results when the loss L_t is set equal to the Dice Score. Variation in performance on the target site between the model as learned on the source only and adapted on the target domain. Dice score on the left, F1 score on the right. On the x-axis is the source center, on the y-axis is the target center.

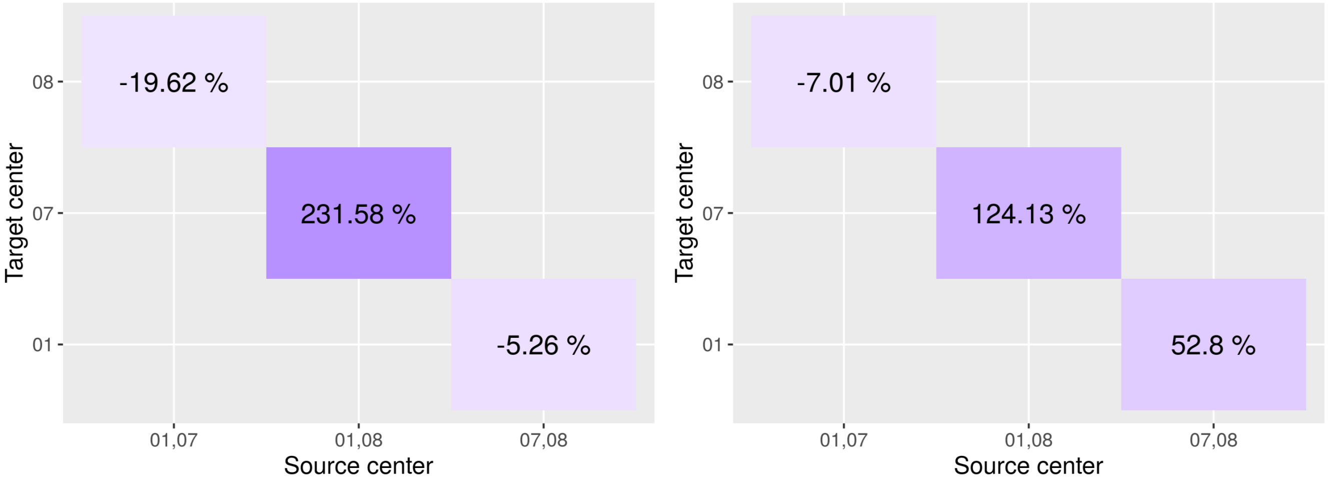

Figure S2. Results when the loss L_t is set equal to the Dice Score. Variation in performance on the target site between the model as learned on the multiple sources and adapted on the target domain. Dice score on the left, F1 score on the right. On the x-axis are the source centers, on the y-axis is the target center.

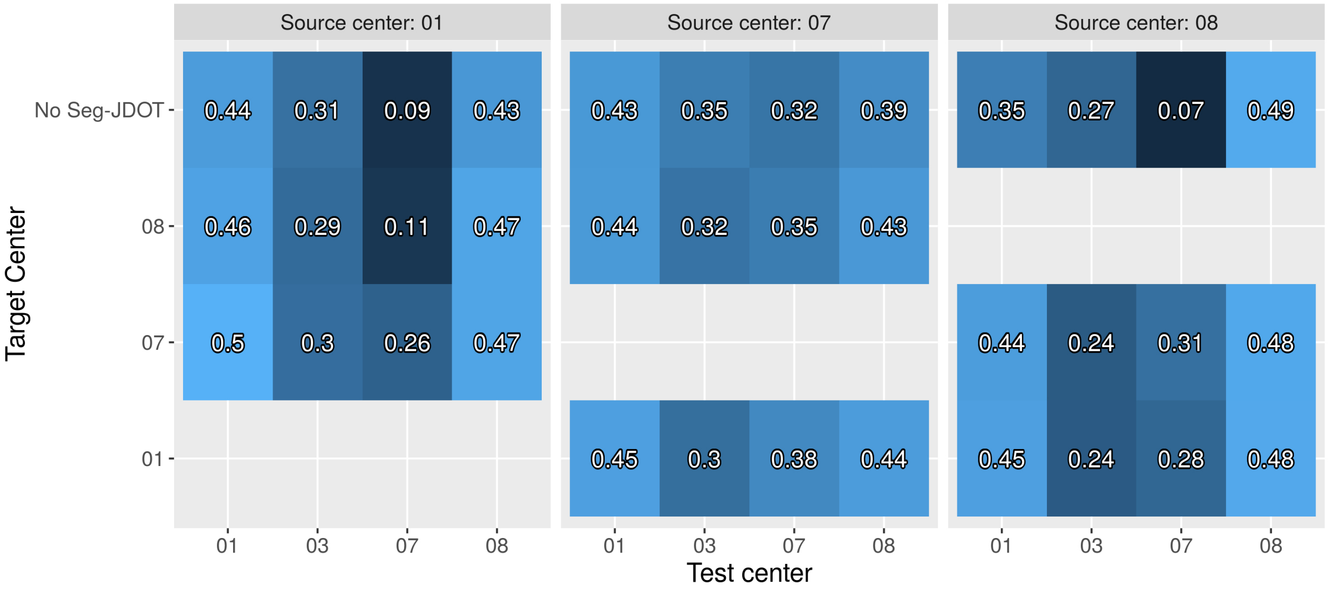

Figure S3. Average Dice score with single-site source and single-site target domain adaptation. The average Dice score is computed with no adaptation (No Seg-JDOT) and with Seg-JDOT, where the direction of the domain adaptation is indicated (07, 08, or 01). For each combination of source and target, performances are given for all the four testing sites.

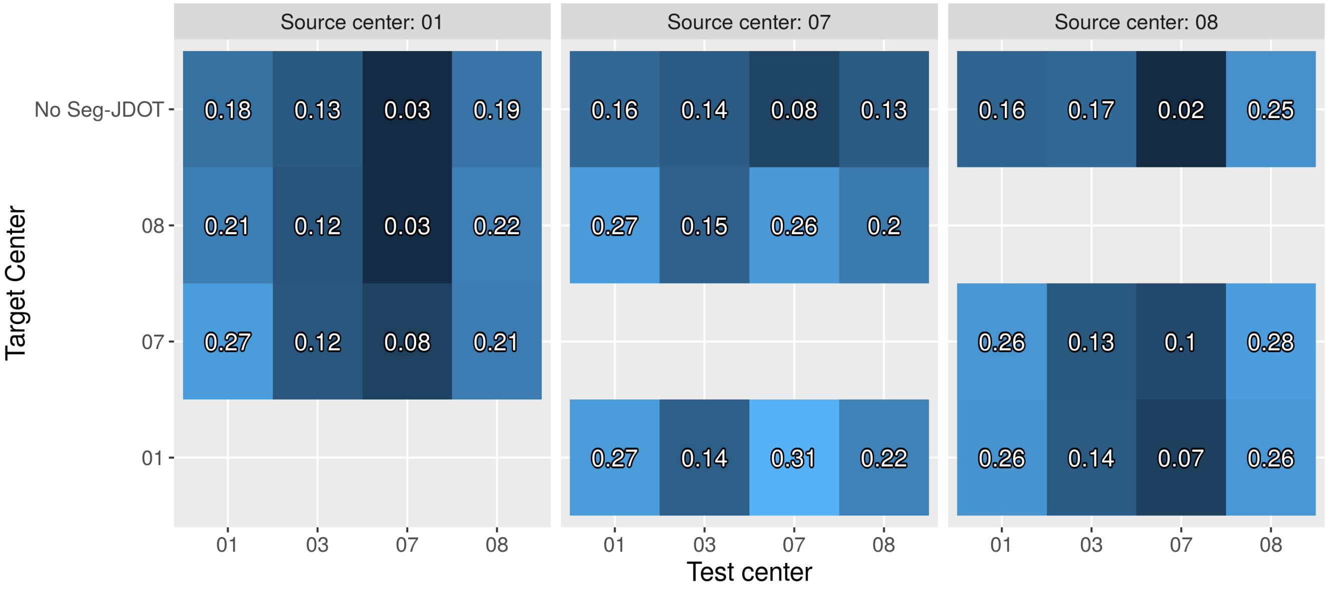

Figure S4. Average F1 score with single-site source and single-site target domain adaptation. The average Dice score is computed with no adaptation (No Seg-JDOT) and with Seg-JDOT, where the direction of the domain adaptation is indicated (07, 08, or 01). For each combination of source and target, performances are given for all the four testing sites.

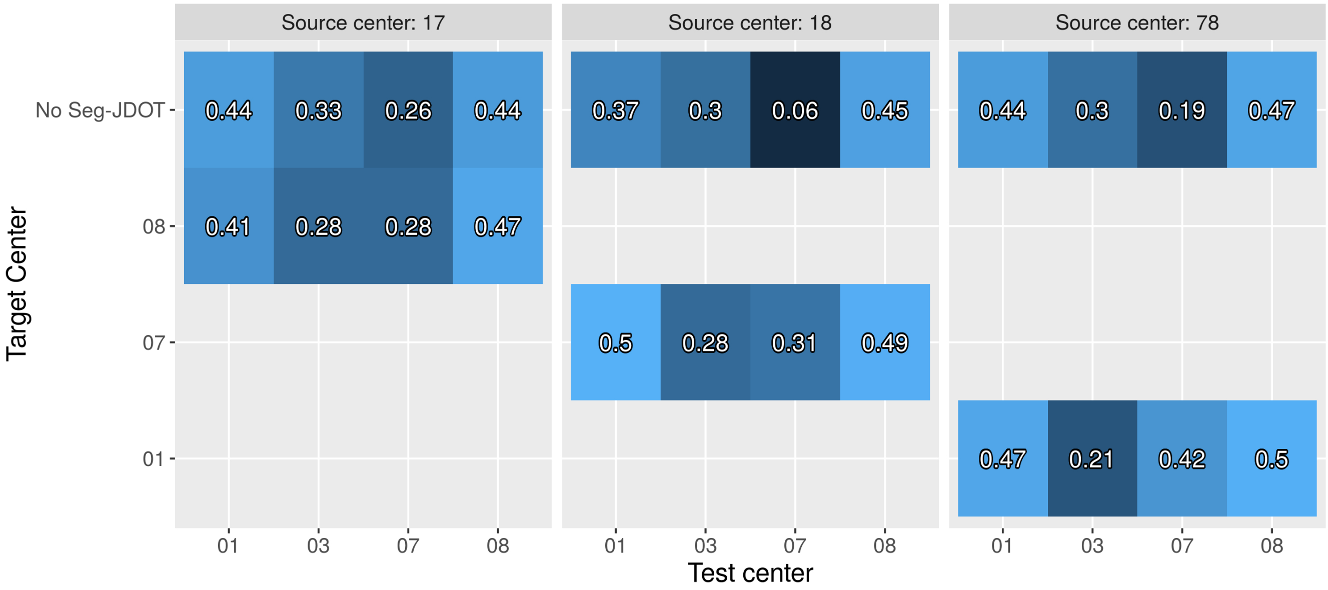

Figure S5. Average Dice score with multi-site source and single-site target domain adaptation. The average Dice score is computed with no adaptation (No Seg-JDOT) and with Seg-JDOT, where the direction of the domain adaptation is indicated (07, 08, or 01). For each combination of source and target, performances are given for all the four testing sites.

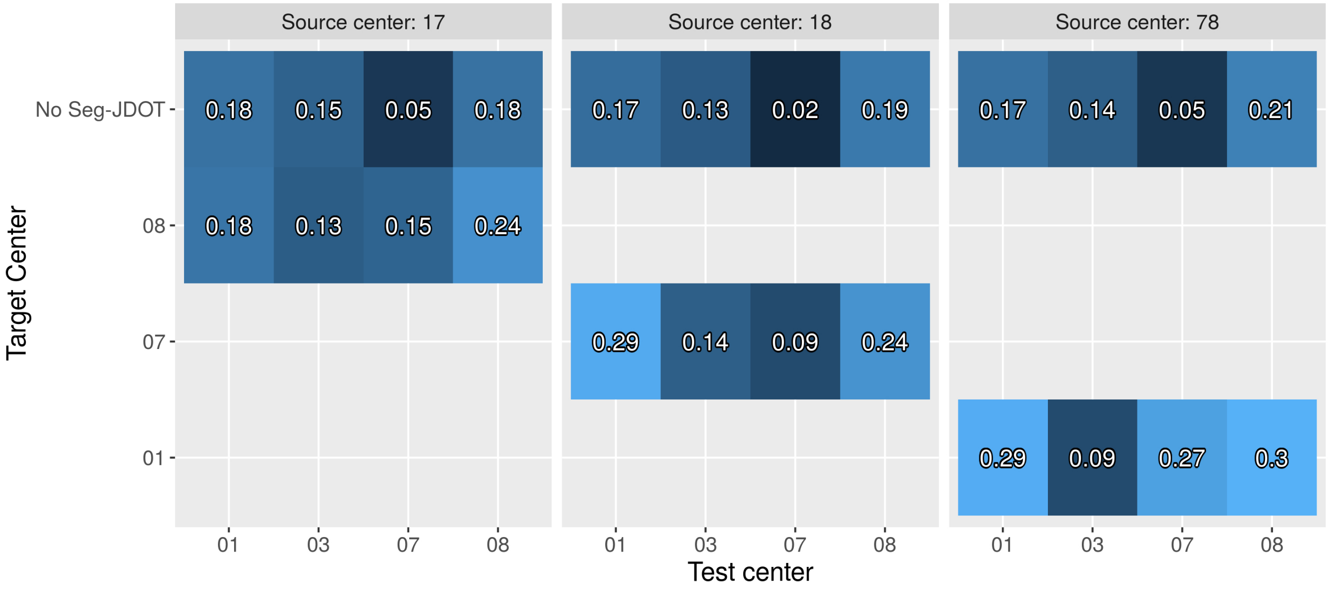

Figure S6. Average F1 score with multi-site source and single-site target domain adaptation. The average Dice score is computed with no adaptation (No Seg-JDOT) and with Seg-JDOT, where the direction of the domain adaptation is indicated (07, 08, or 01). For each combination of source and target, performances are given for all the four testing sites.

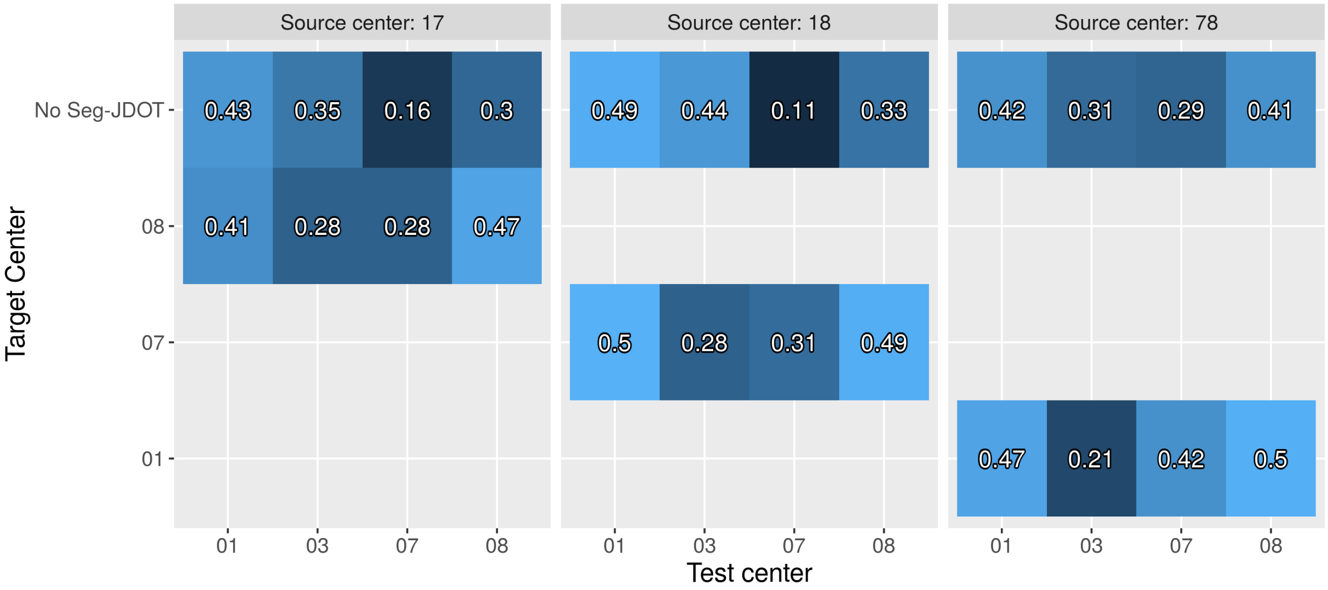

Figure S7. Average Dice score with multi-site source and single-site target domain adaptation. The average Dice score is computed when training on standardized images with no adaptation (No Seg-JDOT) and with Seg-JDOT, where the direction of the domain adaptation is indicated (07, 08, or 01). For each combination of source and target, performances are given for all the four testing sites.

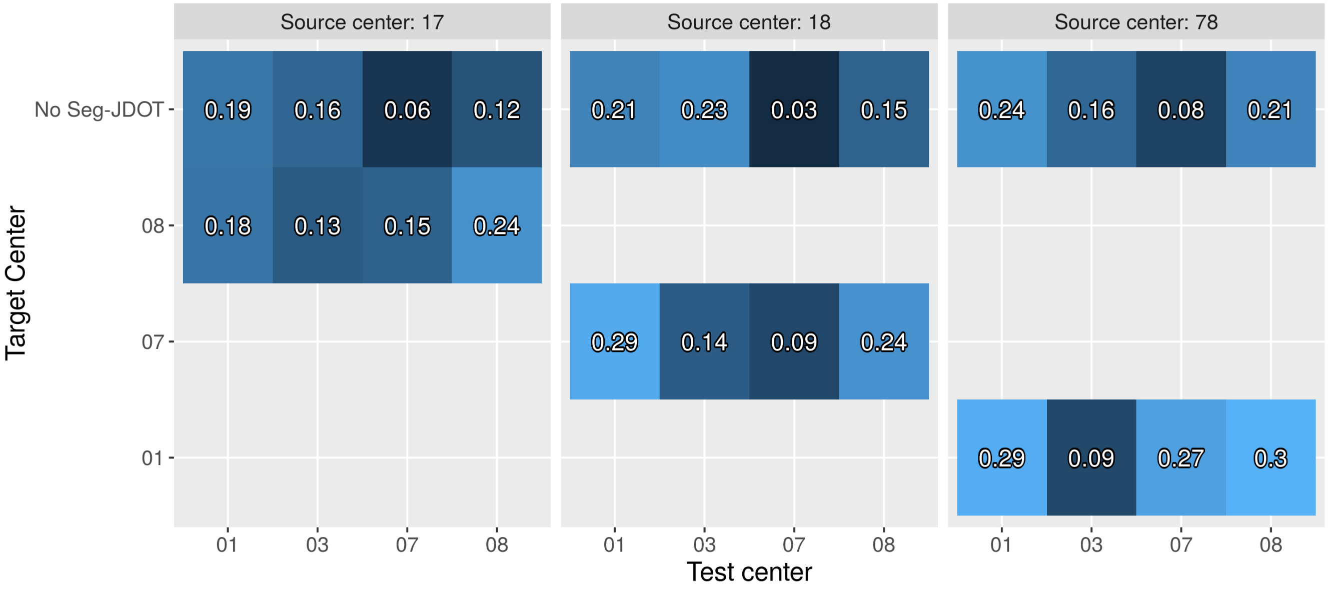

Figure S8. Average F1 score with multi-site source and single-site target domain adaptation. The average Dice score is computed when training on standardized images with no adaptation (No Seg-JDOT) and with Seg-JDOT, where the direction of the domain adaptation is indicated (07, 08, or 01). For each combination of source and target, performances are given for all the four testing sites.

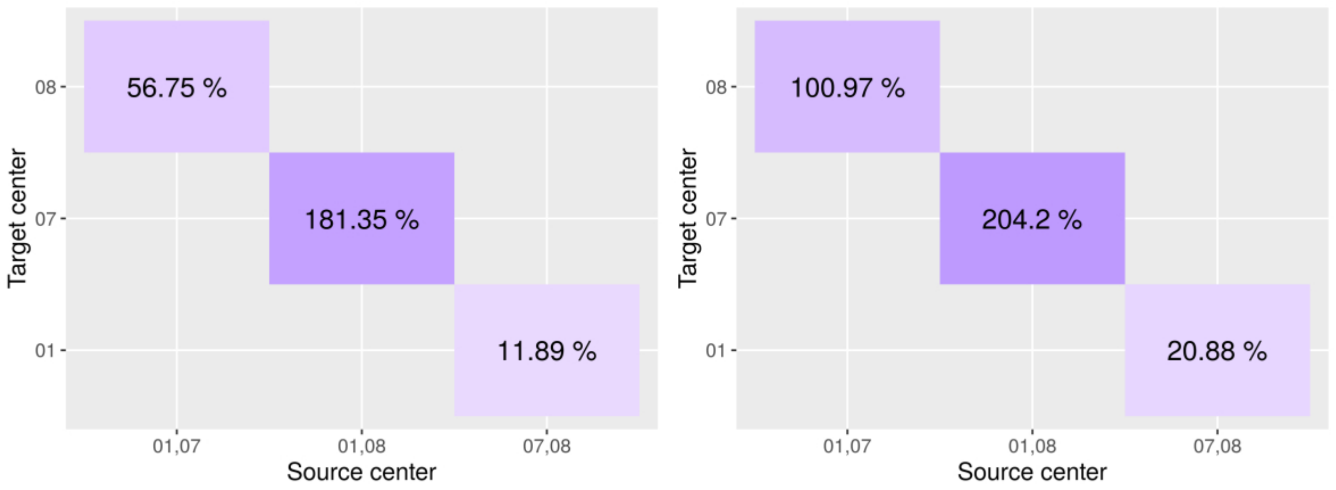

Figure S9. Results after image standardization. Variation in performance on the target site between the model as learned on the multiple sources after image standardization and the model adapted on the target domain. On the x-axis are the source centers, on the y-axis is the target center.
